# Eribulin-induced liver dysfunction as a prognostic indicator of survival of metastatic breast cancer patients: a retrospective study

**DOI:** 10.1186/s12885-016-2436-5

**Published:** 2016-07-07

**Authors:** Takayuki Kobayashi, Jyunichi Tomomatsu, Ippei Fukada, Tomoko Shibayama, Natsuki Teruya, Yoshinori Ito, Takuji Iwase, Shinji Ohno, Shunji Takahashi

**Affiliations:** Department of Medical oncology, Cancer Institute Hospital, 3-8-31 Ariake, Koto-ku, Tokyo, 135-8550 Japan; Department of Breast Medical Oncology, Cancer Institute Hospital, 3-8-31 Ariake, Koto-ku, Tokyo, 135-8550 Japan; Department of Surgical Oncology, Breast Oncology Center, Cancer Institute Hospital, 3-8-31 Ariake, Koto-ku, Tokyo, 135-8550 Japan

**Keywords:** Eribulin-induced liver dysfunction, Fatty liver disease, Metastatic breast cancer

## Abstract

**Background:**

Eribulin is a non-taxane, microtubule dynamics inhibitor that increases survival of patients with metastatic breast cancer. Although eribulin is well tolerated in patients with heavily pretreated disease, eribulin-induced liver dysfunction (EILD) can occur, resulting in treatment modification and subsequent poor disease control. We aimed to clarify the effect of EILD on patient survival.

**Methods:**

The medical records of 157 metastatic breast cancer patients treated with eribulin between July 2011 and November 2013 at Cancer Institute Hospital were retrospectively analyzed. EILD was defined as 1) an increase in alanine aminotransferase or aspartate aminotransferase levels >3 times the upper limit of normal, and/or 2) initiation of a liver-supporting oral drug therapy such as ursodeoxycholic acid or glycyron. Fatty liver was defined as a decrease in the liver-to-spleen attenuation ratio to <0.9 on a computed tomography scan.

**Results:**

EILD occurred in 42 patients, including one patient for whom eribulin treatment was discontinued due to severe EILD. The patients who developed EILD had significantly higher body mass indices (BMIs) than those who did not develop EILD (24.5 vs. 21.5, respectively; *P* < 0.0001), with no difference in the dose intensity of eribulin between the two groups (*P* = 0.76). Interestingly, the patients with EILD exhibited significantly longer progression-free survival (PFS) and overall survival (OS) than those without EILD (*P* = 0.010 and *P* = 0.032, respectively). Similarly, among 80 patients without liver metastasis, 19 with EILD exhibited significantly longer PFS and OS than the others (*P* = 0.0012 and *P* = 0.044, respectively), and EILD was an independent prognostic factor of PFS (*P* = 0.0079) in multivariate analysis. During eribulin treatment, 18 patients developed fatty liver, 11 of whom developed EILD, with a median BMI of 26.7.

**Conclusions:**

Although EILD and fatty liver occurred at a relatively high frequency in our study, most of the patients did not experience severe adverse effects. Surprisingly, the development of EILD was positively associated with patient survival, especially in patients without liver metastases. EILD may be a clinically useful predictive biomarker of survival, but further studies are needed to confirm these findings in another cohort of patients.

## Background

Eribulin mesylate is a synthetic analog of halichondrin B, which is a natural product isolated from the marine sponge *Halichondria okadai*. Eribulin is a non-taxane, microtubule dynamics inhibitor belonging to the halichondrin class of antineoplastic agents [1].

Eribulin was demonstrated to provide significant survival benefits in patients with locally advanced and metastatic breast cancer in a randomized phase III trial that compared the use of eribulin with the treatment of the physician’s choice (TPC) [2], and its use was therefore approved in Japan for breast cancer in July 2011. Currently, it is widely used to treat locally advanced and metastatic breast cancer patients in daily practice.

Although eribulin is well tolerated in patients with heavily pretreated disease [2–5], eribulin-induced liver dysfunction (EILD) can occur in daily practice and may result in treatment modification such as dose-delay, dose-reduction, and treatment discontinuation, leading to poor disease control. Therefore, we conducted a retrospective study to clarify the effects of EILD on the survival of patients with metastatic breast cancer.

## Methods

### Patients

The medical records of 157 metastatic breast cancer patients treated with eribulin at the Cancer Institute Hospital between July 2011 and November 2013 were retrospectively analyzed. This study was approved and the need to obtain informed consent was waived by the Institutional Review Board of Cancer Institute Hospital (2015-1048).

### Definition of EILD and fatty liver

EILD was defined as follows: 1) an increase in alanine aminotransferase (ALT) or aspartate aminotransferase (AST) levels more than three times above the upper limit of normal, and/or 2) initiation or dose-escalation of liver-supporting oral drug therapy, such as ursodeoxycholic acid or glycyron, during eribulin treatment. If patients already had transaminase levels more than 3 times the upper limit of the normal range at the beginning of eribulin treatment, and showed a further increase apparently due to eribulin, they were diagnosed with EILD.

Fatty liver was defined as a liver-to-spleen attenuation ratio of <0.9 on an unenhanced computed tomography (CT) scan [6]. The mean CT attenuation values of the liver and spleen were determined in the parenchyma of the right and left lobes of the liver and of the spleen by using a 100-mm^2^ region of interest cursor, avoiding liver metastases and vessels. CT was performed every 2 or 3 months to evaluate the efficacy of eribulin in most cases.

### Statistical analysis

Comparisons between the treatment groups were evaluated using the chi-square test, Fisher exact test, or Mann-Whitney *U*-test. Progression-free survival (PFS) and overall survival (OS) curves were generated using the Kaplan-Meier method and compared using a log-rank test. Univariate and multivariate Cox proportional hazards models were used to explore the associations of specific clinical variables with PFS and OS. For all tests, differences with *P* < 0.05 were considered statistically significant. All analyses were performed using the JMP 6.0 software package for Windows (SAS Institute Inc., Cary, NC, USA).

## Results

### Patient characteristics and EILD status

The patient characteristics are summarized in Table 1. The median follow-up duration for our cohort was 43.4 weeks (range, 6.0–121.7 weeks). Among the 157 patients treated with eribulin, the median treatment duration was 16.0 weeks (range, 1.9–112.4), the median age was 56 years (range, 25–81), the median disease-free interval was 2.8 years (range, 0–26.5), and the median body mass index (BMI) was 22.2 (range, 15.8–36.3). All patients had a performance status (PS) of 2 or less. Forty-one patients had comorbid diseases including diabetes (*n* = 7), hypertension (*n* = 22), hyperlipidemia (*n* = 6), autoimmune disorders (*n* = 6), cardiovascular disorders (*n* = 6), asthma (*n* = 1), and Parkinson’s disease (*n* = 1). However, these diseases had been controlled well during our observational period. Four patients had a history of other malignancies (2 uterine cervical cancer, one renal cancer, and one Hodgkin lymphoma), but they did not relapse during the follow-up period. Total mastectomy was performed in 142 patients, 35 patients underwent breast-conserving surgery, and 6 patients underwent bilateral breast surgery. A total of 93, 95, and 69 % patients, respectively, had been treated previously with anthracycline, taxane, and capecitabine in the adjuvant or metastatic setting. Among 25 patients with human epidermal growth factor receptor 2 (HER2)-positive tumors, 13 patients had received trastuzumab concurrently with eribulin. Therefore, among the 157 patients treated with eribulin, 144 received eribulin monotherapy. Seventy-seven patients had liver metastases at the start of eribulin treatment.

**Table 1:** Correlation between EILD and clinicopathological variables of the patients treated with eribulin

Variable		Number of cases (%)		
		EILD		
		Yes	No	*P-value*
	Total			
	(*n* = 157)	(*n* = 42)	(*n* = 115)	
Age (years)				
Median (range)	56 (25 ~ 81 y)	57 (25–81 y)	52 (38–73 y)	0.37
PS				
0	104	34	70	0.05
1	41	7	34	
2	12	1	11	
Comorbid disease				
Yes	41	12	29	0.67
Diabetes	7	4	3	
Hypertension	22	9	13	
Hyperlipidemia	6	1	5	
Others	14	2	12	
No	127	31	96	
History of other malignancy				
Yes	4	0	4	0.57
No	153	42	111	
Breast surgery				
Yes	142	39	103	0.75
Total mastectomy	101	25	76	
Breast-conserving surgery	35	13	22	
Bilateral surgery	6	1	5	
No	15	3	12	
DFI (years)				
Median (range)	2.8 (0 ~ 26.5)	3.3 (0–15.6)	2.6 (0–26.5)	0.24
BMI				
Median (range)	22.2 (15.8 ~ 36.3)	24.5 (16.8 ~ 36.3)	21.5 (15.8 ~ 35.4)	<0.0001
HR (ER/PgR) status				
Negative	36	5	31	0.08
Positive	121	37	84	
HER2 status				
Negative	132	37	95	0.56
Positive	25	5	20	
Previous chemotherapy (including adjuvant setting)				
Anthracycline				
Yes	146	40	106	0.73
No	11	2	9	
Taxane				
Yes	149	39	110	0.44
No	8	2	5	
Capecitabine				
Yes	108	25	83	0.13
No	49	17	32	
Number of previous endocrine therapy for metastatic disease				
0	65	14	51	0.37
1	34	14	20	
2	31	7	24	
3	18	4	14	
4	4	2	2	
5	4	1	3	
6	1	0	1	
Type of prior treatment				
Chemotherapy	110	25	85	0.71
Endocrine therapy	42	15	27	
No	5	2	3	
Liver metastasis				
Yes	77	23	54	0.39
No	80	19	61	
Concurrent trastuzumab				
Yes	13	3	10	0.99
No	144	39	105	
Dose intensity of eribulin (mg/m2/week)				
Median (range)	0.72 (0.30 ~ 0.91)	0.72 (0.30–0.90)	0.72 (0.32–0.91)	0.76

Abbreviation: *EILD* eribulin-induced liver dysfunction, *PS* performance status, *DFI* disease free interval, *BMI* body mass index, *HR* hormone receptor, *ER* estrogen receptor, *PgR* progesterone receptor, *HER2* human epidermal growth factor receptor 2

EILD occurred in 42 (27 %) patients, including ten patients who required dose-delays or dose-reductions, and one patient for whom eribulin treatment was discontinued due to EILD. Among the patients with EILD, aminotransferase levels were the highest at a median of 21 days (range, 8–154) after the first eribulin administration. The median AST and ALT levels at the beginning of the eribulin treatment were 34 IU/L (range, 17–78) and 23 IU/L (range, 10–65), respectively, and the medians of the highest AST and ALT levels during eribulin treatment were 93 IU/L (range, 39–300) and 91 IU/L (range, 37–268), respectively.

The patients who developed EILD had significantly higher BMIs than those who did not develop EILD (24.5 vs. 21.5, respectively; *P* < 0.0001; Table 1), while there was no difference in the dose intensity of eribulin between the two groups (0.72 vs. 0.72 mg/m^2^/week, respectively; *P* = 0.76; Table 1). Interestingly, patients with a good PS exhibited a higher frequency of EILD than those with a poor PS (*P* = 0.05, Table 1). The development of EILD was not associated with other clinical factors such as patient age, comorbid disease status, history of other malignancy, primary surgical procedure, disease-free interval (DFI), hormone-receptor (HR) status, HER2 status, previous chemotherapy, previous endocrine therapy, type of prior treatment, and liver metastatic status (Table 1).

### Prognostic value of EILD for patient survival

**Fig. 1:**
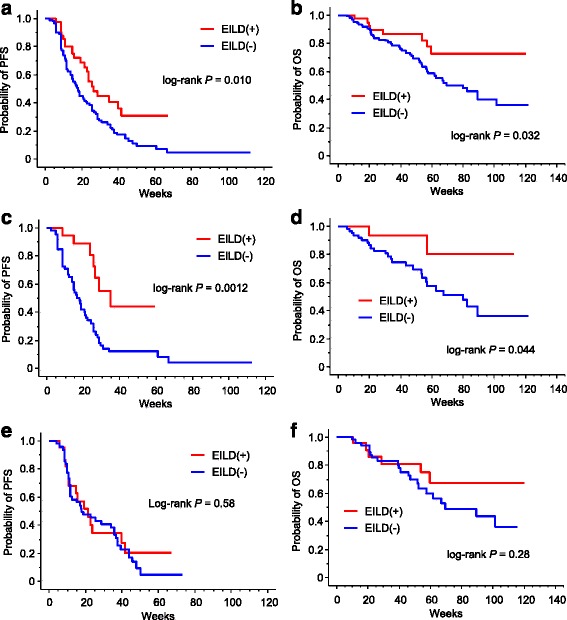
Progression-free survival (PFS) and overall survival (OS) analyses. **a**-**b**: PFS (**a**) and OS (**b**) for patients with and without eribulin-induced liver dysfunction (EILD) among all patients. **c**-**d**: PFS (**c**) and OS (d) for the patients with and without EILD among the patients without liver metastasis. **e**-**f**: PFS (e) and OS (f) for the patients with and without EILD among the patients with liver metastasis

Patients with EILD had significantly longer PFS and OS than those without EILD (*P* = 0.010 and *P* = 0.032, respectively; Fig. 1a and b). Interestingly, this difference appeared to be specific for patients without liver metastases. Among the 80 patients without liver metastases, patients with EILD (*n* = 19) exhibited significantly longer PFS and OS compared patients without EILD (*n* = 61) (*P* = 0.0012 and *P* = 0.044, respectively; Fig. 1c and d). By contrast, EILD was not significantly associated with PFS or OS in patients with liver metastases (*P* = 0.58 and *P* = 0.28, respectively; Fig. 1e and f).

In order to clarify the prognostic role of EILD, we conducted multivariate analyses of clinically significant factors including age, PS, comorbid disease status, DFI, BMI, HR status, HER2 status, liver metastatic status, and development of EILD. In the whole cohort (*n* = 157), multivariate analyses identified only PS as an independent prognostic indicator of better OS (*P* = 0.0039, Table 2), whereas no clinical factors were significantly associated with PFS. In contrast, in the subset of the patients without liver metastases (*n* = 80), EILD was the only significant independent predictor of PFS (*P* = 0.0079, Table 3), and no clinical factors were significantly associated with OS.

**Table 2:** Cox regression analyses for progression-free survival and overall survival in all patients

		Univariate			Multivariate		
		Hazard ratio	(95 % CI)	*P-value*	Hazard ratio	(95 % CI)	*P-value*
Progression-free survival							
EILD	Yes	1.80		0.013	1.44		0.16
	No		1.11–2.88			0.86–2.41	
Age	≦56≦56	0.76		0.14	0.76		0.19
	>56		0.52–1.10			0.51–1.15	
PS	0	1.41		0.089	1.49		0.073
	1,2		0.95–2.10			0.96–2.31	
Comorbid disease	Yes	1.44		0.12	1.51		0.13
	No		0.91–2.28			0.88–2.58	
DFI	≦2.8≦2.8	1.41		0.069	1.27		0.27
	>2.8		0.97–2.06			0.83–1.95	
BMI	>25	1.64		0.036	1.22		0.47
	≦25≦25		1.03–2.59			0.71–2.07	
HR (ER/PgR) status	Positive	1.49		0.062	1.25		0.37
	Negative		0.98–2.28			0.77–2.03	
HER2 status	Negative	1.60		0.067	1.32		0.30
	Positive		0.97–2.65			0.77–2.26	
Liver metastasis	No	0.95		0.067	0.93		0.72
	Yes		0.65–1.38			0.63–1.38	
Overall survival							
EILD	Yes	2.22		0.038	1.75		0.17
	No		1.05–4.71			0.78–3.91	
Age	≦56≦56	0.81		0.45	0.77		0.37
	>56		0.47–1.39			0.43–1.36	
PS	0	2.34		0.0021	2.37		0.0039
	1,2		1.36–4.03			1.32–4.26	
Comorbid disease	Yes	0.91		0.77	1.04		0.91
	No		0.49–1.70			0.51–2.13	
DFI	≦2.8≦2.8	1.78		0.038	1.53		0.18
	>2.8		1.03–3.07			0.83–2.85	
BMI	>25	1.33		0.39	1.13		0.75
	≦25≦25		0.69–2.52			0.55–2.31	
HR (ER/PgR) status	Positive	1.67		0.089	1.15		0.70
	Negative		0.93–3.01			0.57–2.29	
HER2 status	Negative	1.87		0.093	1.42		0.39
	Positive		0.90–3.88			0.64–3.16	
Liver metastasis	No	0.96		0.89	1.00		0.99
	Yes		0.56–1.64			0.56–1.79	

Abbreviation: *95 % CI* 95 % confidence interval, *EILD* eribulin-induced liver dysfunction, *PS* performance status, *DFI* disease free interval, *BMI* body mass index, *HR* hormone receptor, *ER* estrogen receptor, *PgR* progesterone receptor, *HER2* human epidermal growth factor receptor 2

**Table 3:** Cox regression analyses for progression-free survival and overall survival in patients without liver metastasis

		Univariate			Multivariate		
		Hazard ratio	(95 % CI)	*P-value*	Hazard ratio	(95 % CI)	*P-value*
Progression-free survival							
EILD	Yes	3.35		0.0029	3.42		0.0079
	No		1.51–7.44			1.38–8.47	
Age	≦56≦56	0.64		0.11	0.67		0.20
	>56		0.38–1.10			0.36–1.24	
PS	0	0.84		0.58	0.68		0.24
	1,2		0.46–1.55			0.36–1.30	
Comorbid disease	Yes	1.59		0.15	1.47		0.32
	No		0.85–2.99			0.69–3.15	
DFI	≦2.8≦2.8	1.81		0.035	1.79		0.08
	>2.8		1.04–3.15			0.93–3.45	
BMI	>25	1.84		0.073	0.86		0.71
	≦25≦25		0.95–3.57			0.38–1.93	
HR (ER/PgR) status	Positive	1.23		0.46	1.12		0.73
	Negative		0.71–2.13			0.58–2.15	
HER2 status	Negative	1.69		0.14	2.14		0.054
	Positive		0.84–3.39			0.99–4.66	
Overall survival							
EILD	Yes	3.93		0.063	3.42		0.13
	No		0.93–16.6			0.71–16.44	
Age	≦56≦56	0.81		0.59	0.89		0.78
	>56		0.38–1.73			0.38–2.09	
PS	0	1.73		0.20	1.51		0.36
	1,2		0.75–4.02			0.63–3.63	
Comorbid disease	Yes	1.14		0.76	1.17		0.76
	No		0.48–2.71			0.44–3.09	
DFI	≦2.8≦2.8	2.52		0.037	1.95		0.19
	>2.8		1.06–5.98			0.73–5.25	
BMI	>25	1.47		0.42	0.87		0.79
	≦25≦25		0.59–3.62			0.32–2.40	
HR (ER/PgR) status	Positive	1.54		0.28	0.97		0.95
	Negative		0.71–3.34			0.42–2.28	
HER2 status	Negative	2.49		0.057	2.20		0.13
	Positive		0.97–6.39			0.79–3.09	

Abbreviation: *95 % CI* 95 % confidence interval, *EILD* eribulin-induced liver dysfunction, *PS* performance status, *DFI* disease free interval, *BMI* body mass index, *HR* hormone receptor, *ER* estrogen receptor, *PgR* progesterone receptor, *HER2* human epidermal growth factor receptor 2

### Fatty liver disease during eribulin treatment

During eribulin treatment, 18 (11 %) patients who developed fatty liver disease had a median BMI of 26.7, which was significantly higher than that of patients who did not develop fatty liver disease (26.7 vs. 21.7, respectively; *P* < 0.0001). Eleven (61 %) of these 18 patients developed EILD.

## Discussion

In the present study, we found that EILD occurred with a relatively high frequency, but most patients, except one, did not experience severe liver damage. In this particular patient, eribulin treatment was terminated because the patient’s aminotransferase levels increased to more than ten times the upper limit of normal and PS decreased to and remained at 3 even after aminotransferase levels peaked in the early phase of eribulin treatment. While liver toxicity was uncommon in the global phase III trial of eribulin vs. TPC, in which 93 % of the patients were Caucasian [2], a Japanese phase II trial demonstrated liver toxicity in approximately 30 % of patients [5]. These studies suggest that ethnicity may influence eribulin-induced liver toxicity.

To our knowledge, this is the first study to demonstrate that eribulin induces fatty liver disease, and that the development of both EILD and fatty liver disease is significantly associated with higher BMI. These results indicate that eribulin might precipitate liver damage and latent fatty liver in patients with obesity. The main mechanism of chemotherapy-induced liver injury is thought to be secondary to the production of reactive oxygen species (ROS), and steatotic livers are more susceptible to chemotherapy-induced injury [7]. Presumably, eribulin might also produce ROS in hepatocytes in a similar manner, resulting in EILD and fatty liver disease. In fact, other microtubule-targeted agents such as paclitaxel and vinorelbine were shown to induce accumulation of ROS in cancer cell lines, and their anti-tumor effects partially depend on this mechanism [8, 9].

Paradoxically, we observed a positive correlation between the development of EILD and patient survival, especially in patients without liver metastases. Therefore, EILD may be a clinically useful and easily available biomarker that can be used to predict the efficacy of eribulin in the early stages of treatment. One possible reason for this finding may be that patients with EILD had a better nutritional status, and hence, they could survive longer. However, previous studies showed that obesity was a poor prognostic factor for patients with metastatic breast cancer [10, 11]. Furthermore, most adjuvant clinical trials showed the same results [12].

Another possible cause of our paradoxical results is that eribulin-induced ROS production might result in the eradication of minute metastases consisting mainly of cancer stem cells (CSCs). The CSC hypothesis has been widely accepted, and CSCs are considered to play an important role in the initiation of tumor metastasis [13–16]. Moreover, ROS have a dual role in cancer progression. Although ROS are thought to play an important role in carcinogenesis initiation, malignant transformation, and cell proliferation, excess ROS production can also trigger apoptosis of malignant cells [17]. Cellular ROS metabolism is tightly regulated by the redox mechanism, and ROS concentrations are maintained lower especially in CSCs compared to non-CSCs [18–20]. ROS elevation by exogenous drugs may be a potential treatment strategy to selectively kill CSCs [21], and in fact, some chemotherapeutic drugs have been shown to elicit such an effect on leukemic stem cells [22–24].

Taken together, these previous evidences described above support our hypothesis. In fact, we found that, among the 70 patients without liver metastasis at the beginning of eribulin treatment who were evaluated for liver metastasis at the final follow-up, the appearance of new metastatic liver lesions was less frequent in those who developed EILD than in those who did not (2 of 19 [10.5 %] and 12 of 51 [23.5 %], respectively). This is consistent with our hypothesis that eribulin-induced ROS production eradicates minute disseminated CSCs in the liver. However, the difference between these frequencies was not significant (*P* = 0.32); therefore, larger studies are needed to further evaluate this hypothesis.

## Conclusions

In summary, although EILD occurred with a relatively high frequency in eribulin-treated breast cancer patients, it was generally well tolerated in heavily pretreated patients in clinical practice. To our knowledge, this is the first study to show that eribulin may induce fatty liver disease, and that EILD and fatty liver disease occur more frequently in obese patients.

We found that EILD was a significant positive prognostic factor for breast cancer patient survival, especially among patients without liver metastasis. EILD may be a clinically useful and easily available biomarker that can be used to predict the efficacy of eribulin in the early stages of treatment. However, our study was limited by its small size, retrospective design, and restriction to a single institute. Therefore, further studies are needed to confirm our findings in other patient cohorts and to elucidate the mechanism of EILD.

## Abbreviations

BMI, body mass index; CSC, cancer stem cell; CT, computed tomography; DFI, disease-free survival; EILD, eribulin-induced liver dysfunction; HER2, human epidermal growth factor receptor 2; HR, hormone receptor; OS, overall survival; PFS, progression-free survival; TPC, treatment of physician’s choice
